# Supplementary material for: Genome-wide assessment of population structure and genetic diversity of Chinese Lou onion using specific length amplified fragment (SLAF) sequencing
Source: PLoS One. 2020 May 5;15(5):e0231753. doi: 10.1371/journal.pone.0231753 (PMC7199963; doi:10.1371/journal.pone.0231753)
Supplement: S1 Table — (DOCX) [file pone.0231753.s001.docx]

**Table S1. The original data of biophysical traits in *A.fistulosum* L. var. *viviparum* Makino from different sites.**

| **Sites** | **Site Name** | **Individual ID** | **Pseudostem Firmness (g/cm^2^)** | **Dry matter rate (B2,%)** | **Plant weight (g)** | **Plant Height (cm)** | **Pseudostem Weight (g)** | **Pseudostem Length (cm)** | **Pseudostem Diameter (cm)** | **Follower height (cm)** | **Pseudostem**  **index** |
| --- | --- | --- | --- | --- | --- | --- | --- | --- | --- | --- | --- |
| 1 | Datong, Qinghai | 1-1 | 0.56 | 9.32 | 97.12 | 46.25 | 72.23 | 12.5 | 2.36 | 31 | 5.29 |
|  |  | 1-2 | 0.56 | 9.33 | 89.23 | 49 | 61.25 | 12.75 | 2.56 | 33.75 | 4.99 |
|  |  | 1-3 | 0.55 | 9.27 | 90.42 | 47 | 68.33 | 12 | 1.6 | 31 | 7.5 |
| 2 | Bailang, Tibet | 2-1 | 0.49 | 6.27 | 80.19 | 48.5 | 56.23 | 13.5 | 1.89 | 35 | 7.15 |
|  |  | 2-2 | 0.47 | 6.06 | 81.32 | 50.5 | 55.55 | 12 | 1.92 | 31.5 | 6.26 |
|  |  | 2-3 | 0.49 | 6.25 | 79.24 | 50.5 | 53.25 | 13.5 | 1.35 | 33 | 9.99 |
| 3 | Linzhi, Tibet | 3-1 | 0.55 | 10.27 | 87.19 | 53.25 | 68.32 | 14.25 | 1.82 | 35.25 | 7.83 |
|  |  | 3-2 | 0.59 | 10.03 | 89.32 | 56 | 69.17 | 14 | 1.74 | 38.75 | 8.06 |
|  |  | 3-3 | 0.59 | 10.87 | 89.24 | 59.75 | 68.54 | 14.75 | 3.13 | 41 | 4.72 |
| 4 | Baode,Shanxi | 4-1 | 0.63 | 9.27 | 97.67 | 66.5 | 67.67 | 15.5 | 2.36 | 47 | 6.57 |
|  |  | 4-2 | 0.63 | 9.03 | 99.44 | 62 | 69.44 | 13 | 2.01 | 42 | 6.46 |
|  |  | 4-3 | 0.63 | 9.87 | 96.92 | 72 | 66.92 | 11 | 1.81 | 45.5 | 6.09 |
| 5 | Hequ,Shanxi | 5-1 | 0.65 | 10.06 | 101.1 | 54 | 71.1 | 14 | 2.53 | 38 | 5.53 |
|  |  | 5-2 | 0.67 | 10.08 | 99.89 | 57 | 69.89 | 13.5 | 3 | 38 | 4.5 |
|  |  | 5-3 | 0.66 | 10.05 | 100.21 | 62 | 70.21 | 12.5 | 2.11 | 43 | 5.92 |
| 6 | Zhongwei,Ningxia | 6-1 | 0.68 | 10.97 | 132.43 | 71.25 | 102.43 | 16.75 | 2.76 | 47 | 6.07 |
|  |  | 6-2 | 0.68 | 10.76 | 135.12 | 69.5 | 105.12 | 14 | 2.43 | 46.5 | 5.77 |
|  |  | 6-3 | 0.69 | 10.59 | 139.15 | 66 | 109.15 | 12.5 | 2.04 | 46.5 | 6.13 |
| 7 | Jiaxian, Shaanxi | 7-1 | 0.52 | 8.28 | 109.19 | 60.5 | 79.19 | 12.5 | 2.43 | 40.5 | 5.15 |
|  |  | 7-2 | 0.5 | 8.37 | 104.56 | 62 | 74.56 | 14.5 | 1.58 | 40 | 9.16 |
|  |  | 7-3 | 0.52 | 8.29 | 107.25 | 63 | 77.25 | 13 | 2.35 | 43 | 5.53 |
| 8 | Mizhi,Shaanxi | 8-1 | 0.55 | 9.06 | 109.54 | 62.5 | 79.54 | 14.5 | 2.31 | 41.5 | 6.27 |
|  |  | 8-2 | 0.57 | 9.18 | 108.91 | 61.5 | 78.91 | 13.5 | 2.48 | 42 | 5.43 |
|  |  | 8-3 | 0.58 | 9.48 | 107.96 | 66.5 | 77.96 | 16.5 | 2.08 | 44.5 | 7.94 |
| 9 | Yanchi,Ningxia | 9-1 | 0.63 | 10.15 | 141.08 | 72.5 | 111.08 | 16 | 2.85 | 49.5 | 5.61 |
|  |  | 9-2 | 0.63 | 10.19 | 143.37 | 71.5 | 113.37 | 14.5 | 1.85 | 46.5 | 7.84 |
|  |  | 9-3 | 0.65 | 10.23 | 140.42 | 70 | 110.42 | 15.5 | 2.36 | 46.5 | 6.57 |
| 10 | Zhongning,Ningxia | 10-1 | 0.64 | 10.79 | 140.08 | 71 | 110.08 | 16 | 2.61 | 43.75 | 6.13 |
|  |  | 10-2 | 0.65 | 10.97 | 141.37 | 69.5 | 111.37 | 15.75 | 2.56 | 44.25 | 6.15 |
|  |  | 10-3 | 0.64 | 10.95 | 139.42 | 68.5 | 109.42 | 16.25 | 2.61 | 44.75 | 6.22 |
| 11 | Tongxin-1, Ningxia | 11-1 | 0.84 | 19.79 | 153.08 | 78 | 113.08 | 13.5 | 2.44 | 53 | 5.52 |
|  |  | 11-2 | 0.85 | 19.27 | 161.37 | 75.5 | 111.37 | 17.5 | 3.08 | 48.5 | 5.69 |
|  |  | 11-3 | 0.87 | 19.03 | 153.42 | 74.5 | 113.42 | 14.5 | 3.37 | 49 | 4.3 |
| 12 | Hetao, Inner mongolia | 12-1 | 0.58 | 8.48 | 112.43 | 77.6 | 82.43 | 18.7 | 1.61 | 50 | 11.59 |
|  |  | 12-2 | 0.58 | 9.14 | 115.12 | 77.3 | 85.12 | 18.5 | 1.38 | 51.25 | 13.4 |
|  |  | 12-3 | 0.59 | 8.96 | 119.15 | 74.5 | 89.15 | 18 | 1.23 | 45.75 | 14.58 |
| 13 | Xiji,Ningxia | 13-1 | 0.74 | 12.14 | 135.77 | 61.25 | 105.77 | 14.25 | 3.06 | 41.4 | 4.65 |
|  |  | 13-2 | 0.74 | 11.99 | 135.36 | 54.5 | 105.36 | 15 | 3.1 | 44.75 | 4.84 |
|  |  | 13-3 | 0.74 | 12.02 | 133.48 | 59 | 103.48 | 16 | 2.27 | 39 | 7.05 |
| 14 | Yinchuan,Ningxia | 14-1 | 0.62 | 10.14 | 129.51 | 62.25 | 99.51 | 15 | 3.13 | 42.75 | 4.79 |
|  |  | 14-2 | 0.63 | 10.15 | 128.49 | 60 | 98.49 | 16.25 | 3.22 | 37.75 | 5.05 |
|  |  | 14-3 | 0.63 | 10.19 | 122.98 | 63.25 | 92.98 | 16 | 2.16 | 42.25 | 7.41 |
| 15 | Tongxin-2, Ningxia | 15-1 | 0.9 | 20.27 | 159.78 | 79.5 | 119.78 | 18.5 | 2.1 | 52 | 8.81 |
|  |  | 15-2 | 0.88 | 20.08 | 155.67 | 78.25 | 115.67 | 16.5 | 3.91 | 51.5 | 4.22 |
|  |  | 15-3 | 0.89 | 20.18 | 159.42 | 79 | 119.42 | 18.5 | 2.74 | 55 | 6.74 |
| 16 | Wuwei,Gansu | 16-1 | 0.72 | 14.27 | 136.78 | 69.5 | 106.78 | 15.25 | 2.45 | 45.5 | 6.22 |
|  |  | 16-2 | 0.72 | 13.98 | 137.32 | 70.25 | 107.32 | 15.5 | 2.48 | 45.25 | 6.26 |
|  |  | 16-3 | 0.72 | 14.11 | 140.56 | 69 | 110.56 | 15.25 | 2.65 | 49 | 5.75 |
| 17 | Haiyuan,Ningxia | 17-1 | 0.71 | 11.14 | 133.87 | 75.25 | 103.87 | 17.75 | 3.16 | 50 | 5.62 |
|  |  | 17-2 | 0.72 | 11.55 | 135.62 | 75.25 | 105.62 | 18 | 4.43 | 51.25 | 4.07 |
|  |  | 17-3 | 0.71 | 11.67 | 133.56 | 66 | 103.56 | 16 | 3.19 | 45.75 | 5.02 |
| 18 | Longde,Ningxia | 18-1 | 0.68 | 11.04 | 136.65 | 73.25 | 106.65 | 36.5 | 3.47 | 46.5 | 10.52 |
|  |  | 18-2 | 0.63 | 10.97 | 129.49 | 71.25 | 109.49 | 17.5 | 3.98 | 45.75 | 4.4 |
|  |  | 18-3 | 0.65 | 10.45 | 133.67 | 67 | 103.67 | 15.25 | 4.12 | 45.5 | 3.7 |
| 19 | Zizhou,Shaanxi | 19-1 | 0.52 | 8.67 | 101.43 | 56 | 71.43 | 15 | 2.03 | 38.5 | 7.39 |
|  |  | 19-2 | 0.53 | 8.73 | 107.22 | 59.5 | 77.22 | 17.5 | 2.47 | 40.25 | 7.09 |
|  |  | 19-3 | 0.53 | 8.88 | 102.56 | 58.5 | 72.56 | 12 | 1.84 | 37 | 6.51 |
| 20 | Tacheng,Tibet | 20-1 | 0.62 | 9.25 | 119.43 | 71.45 | 89.43 | 17.15 | 2.86 | 52 | 6 |
|  |  | 20-2 | 0.6 | 8.87 | 121.12 | 72.15 | 91.12 | 17 | 2.43 | 53.25 | 7 |
|  |  | 20-3 | 0.62 | 9.17 | 122.15 | 71.25 | 92.15 | 16.5 | 2.29 | 49.75 | 7.21 |
| 21 | Yumin, Tibet | 21-1 | 0.61 | 10.11 | 121.43 | 72.45 | 91.43 | 17.75 | 2.7 | 48.9 | 6.58 |
|  |  | 21-2 | 0.62 | 10.15 | 122.12 | 72.35 | 92.12 | 17 | 2.5 | 47.25 | 6.8 |
|  |  | 21-3 | 0.63 | 10.19 | 124.15 | 70.9 | 94.15 | 16.75 | 2.27 | 43 | 7.39 |
| 22 | Jingbian,Shaanxi | 22-1 | 0.57 | 9.33 | 108.77 | 68 | 78.77 | 14.5 | 2.05 | 48 | 7.09 |
|  |  | 22-2 | 0.57 | 9.38 | 106.65 | 69.5 | 76.65 | 15.5 | 1.92 | 53 | 8.07 |
|  |  | 22-3 | 0.59 | 9.29 | 107.27 | 68.5 | 77.27 | 17 | 3.23 | 45.5 | 5.26 |
| 23 | Baiyin,Gansu | 23-1 | 0.71 | 14.17 | 135.28 | 68.75 | 105.28 | 14.25 | 2.61 | 42.4 | 5.45 |
|  |  | 23-2 | 0.71 | 13.78 | 135.77 | 70.75 | 105.77 | 15 | 2.6 | 44.5 | 5.77 |
|  |  | 23-3 | 0.71 | 14.22 | 133.62 | 72.75 | 103.62 | 15 | 2.27 | 44 | 6.61 |
