# Supplementary material for: Genome-wide assessment of population structure and genetic diversity of Chinese Lou onion using specific length amplified fragment (SLAF) sequencing
Source: PLoS One. 2020 May 5;15(5):e0231753. doi: 10.1371/journal.pone.0231753 (PMC7199963; doi:10.1371/journal.pone.0231753)
Supplement: Attachment — Submitted filename: Response to Reviewers.docx [file pone.0231753.s004.docx]

Dear Tzen-Yuh Chiang,

Academic Editor

PLOS ONE,

We are responding to the editor and reviewers’ comments concerning our manuscript entitled “Genome-wide assessment of population structure and genetic diversity of Chinese Lou onion using specific length amplified fragment (SLAF) sequencing” (ID: PONE-D-19-22783).

Thank you for your letter and for the comments which are all valuable and very helpful for revising and improving our paper. We have studied the comments carefully and have made corrections as marked in the revisions and listed as follows:

**Point-by-point response to the reviewer’s comments**

Journal Requirements:

When submitting your revision, we need you to address these additional requirements.

Please ensure that your manuscript meets PLOS ONE's style requirements, including those for file naming. The PLOS ONE style templates can be found at

<http://www.journals.plos.org/plosone/s/file?id=wjVg/PLOSOne_formatting_sample_main_body.pdf> and <http://www.journals.plos.org/plosone/s/file?id=ba62/PLOSOne_formatting_sample_title_authors_affiliations.pdf>

1. In your Data Availability statement, you have not specified where the minimal data set underlying the results described in your manuscript can be found. PLOS defines a study's minimal data set as the underlying data used to reach the conclusions drawn in the manuscript and any additional data required to replicate the reported study findings in their entirety. All PLOS journals require that the minimal data set be made fully available. For more information about our data policy, please see <http://journals.plos.org/plosone/s/data-availability.>

Upon re-submitting your revised manuscript, please upload your study’s minimal underlying data set as either Supporting Information files or to a stable, public repository and include the relevant URLs, DOIs, or accession numbers within your revised cover letter. For a list of acceptable repositories, please see <http://journals.plos.org/plosone/s/data-availability#loc-recommended-repositories.> Any potentially identifying patient information must be fully anonymized.

Important: If there are ethical or legal restrictions to sharing your data publicly, please explain these restrictions in detail. Please see our guidelines for more information on what we consider unacceptable restrictions to publicly sharing data: <http://journals.plos.org/plosone/s/data-availability#loc-unacceptable-data-access-restrictions.> Note that it is not acceptable for the authors to be the sole named individuals responsible for ensuring data access.

We will update your Data Availability statement to reflect the information you provide in your cover letter.

**-Response:** The original data has been supplied as Supplementary Table S1.

2. Please include your tables as part of your main manuscript and remove the individual files. Please note that supplementary tables (should remain/ be uploaded) as separate "supporting information" files
**-Response:** We attached the table 1 below the main text according to the comment.

[Note: HTML markup is below. Please do not edit.]

Reviewers' comments:

Reviewer's Responses to Questions

**Comments to the Author**

1. Is the manuscript technically sound, and do the data support the conclusions?

The manuscript must describe a technically sound piece of scientific research with data that supports the conclusions. Experiments must have been conducted rigorously, with appropriate controls, replication, and sample sizes. The conclusions must be drawn appropriately based on the data presented.

Reviewer #1: Partly

Reviewer #2: Partly

**-Response:** The results, especial Table 1, have been well displayed and discussed according to the comments from the two reviewers.

2. Has the statistical analysis been performed appropriately and rigorously?

Reviewer #1: Yes

Reviewer #2: Yes

**-Response:** Thanks for your comments.

3. Have the authors made all data underlying the findings in their manuscript fully available?

The [PLOS Data policy](http://www.plosone.org/static/policies.action#sharing)requires authors to make all data underlying the findings described in their manuscript fully available without restriction, with rare exception (please refer to the Data Availability Statement in the manuscript PDF file). The data should be provided as part of the manuscript or its supporting information, or deposited to a public repository. For example, in addition to summary statistics, the data points behind means, medians and variance measures should be available. If there are restrictions on publicly sharing data—e.g. participant privacy or use of data from a third party—those must be specified.

Reviewer #1: No

Reviewer #2: Yes

**-Response:** The original data for table 1 has been supplied as Supplementary Table S1.

4. Is the manuscript presented in an intelligible fashion and written in standard English?

PLOS ONE does not copyedit accepted manuscripts, so the language in submitted articles must be clear, correct, and unambiguous. Any typographical or grammatical errors should be corrected at revision, so please note any specific errors here.

Reviewer #1: Yes

Reviewer #2: Yes

**-Response:** Thanks for your comments. The manuscript has been improved according to other comments. However, the minor errors in grammar and writing have been checked and revised now.

5. Review Comments to the Author
Please use the space provided to explain your answers to the questions above. You may also include additional comments for the author, including concerns about dual publication, research ethics, or publication ethics. (Please upload your review as an attachment if it exceeds 20,000 characters)

**Reviewer #1:**

In this manuscript, the authors described their findings about the genetic diversity of the Luo onion in China. As onion is one of the most widely grown agricultural crop, this study has some merits in understanding the genetic background of onions grown in different places in China. However, the authors only described some numbers superficially with few interpretations, and the authors did not include enough details about what they have done, making the manuscripts they wrote seems too simple. Even the figure legends are over simple, making it hard to know the points the they want to make. Thus, I suggest the authors to add more interpretation, spend some time thinking about what to illustrate in the paper and write it more careful. Below are some suggestions.

**-Response:** Thanks for your comments and suggestions. We have made revisions or modifications according to your kind suggestions as listed below.

Deposit the sequenced reads to public databases such as NCBI SRA.

**-Response:** The raw sequence data reported in this paper have been deposited in the Genome Sequence Archive in BIG Data Center, Beijing Institute of Genomics (BIG), Chinese Academy of Sciences, under submit accession number subCRA002329 that are publicly accessible at <https://bigd.big.ac.cn/gsa>.

In the method part, the authors mentioned that “Statistical analysis was performed using the one-way ANOVA”. However, the authors did not mention where they do the test.

**-Response:** The methods were revised to “Statistical analysis was performed using the one-way ANOVA followed with LSD test in SPSS 22.0” according to your suggestion. SPSS 22.0 was used for statistical analysis.

It is in Table 1 when they were comparing the biological traits of varietas collected from different place. The authors may add more description about how they controlled the growing of those onions and how many samples they tested in each position to get the values for those traits.

**-Response:** Thank you for your comments, the sampling details were added in the newly submitted manuscript. Onions were placed in field fertilized with 6 m3/667 m2 commercial organic fertilizer (45% organic matter, 5% N+P2O5+K2O, 20 million viable bacteria per gram) with micro-spray irrigation (1500 m3/hm2 water per year).

The numbers in table 1 seem too perfect to be true. I suggest the authors to add a supplement to include the original numbers they got.

**-Response:** The original data is supplied as Table S1. We promise here that all the data is true.

Table 1, “a-f, different letters in the same line indicate a significant different between treatments”. This description is wrong.

**-Response:** We changed it to “Statistically significant differences (p<0.05) between sampling sites are noted by different letters within a column”.

Also, while most values have the significant group letter as superscript, some are not.

**-Response:** Thanks for your careful review. The first two letters in the first column have been changed to superscript now.

What are the row “P” and the row “F”? What is “DR”? “Dunnett’3 test” should be “Dunnett’s test”. Which one you use LSD test and which “Dunnett’s test”?

**-Response:** Sorry for the wrong writing. We perform all the statistical analyses with the LSD test.

Line 125, “Table 1 showed that the accessions from the two sites at Tongxi had relative higher dry matter rate and firmness, plant weight and height, pseudostem weight, length and diameter, and follow height”. The authors made the LSD test but did not draw conclusion based on the test.

**-Response:** Thanks for your comment. This part has been revised and the related sentences are “For instance, the pseudostem firmness of accessions from Tongxin, Ningxia was 0.85±0.02 g/cm2 and 0.89±0.01 g/cm2, respectively, which was insignificantly higher than 0.74±0.00% (Xiji, Ningxia), 0.71±0.01% (Haiyuan, Ningxia), 0.72±0.00% (Wuwei, Gansu) and 0.71±0.00% (Baiyin,Gansu) of accessions from Ningxia and Gansu, but were significantly higher than <0.68±0.01% from others. The dry matter rate of the accessions from Tongxin, Ningxia was 20.18±0.09% and 19.36±0.39%, respectively, which were significantly higher than < 14.06±0.24% (14.12±0.15, Wuwei,Gansu) from other accessions. The accessions from Hetao, Inner mongolia had the highest pseudostem index (13.19±1.51) compared with others (less than 7.80±1.95, p < 0.05).”

Line 127, “there was not difference”, change to “there was no significant difference”.

**-Response:** This sentence has been changed as aforementioned. There are differences in the pseudostem index between sites.

Again, I doubted the results. It is hard for a value like “13.19±1.51” not significantly different from other values in the “pseudostem index” column of Table 1. I strongly suggest the authors to include the original data for the test.

**-Response:** Sorry for the missing analysis. We have analyzed the pseudostem index and the difference is presented in Table 1. Thanks for your careful review and the valuable correction. The original data was provided at your invitation (Supplementary Table S1).

Fig. 1, use English names provinces in the figure. What are those arrows? How many samples were collected in each place and what are those samples? Which groups the samples belong to in Figure 4B? “Red star indicates the capital city of China, Beijing”. What point you want to make from this description? Help people understand the geography of China?

**-Response:** Thank you for your comments. Figure 1 was reversed in the newly submitted manuscript. And the names of the sampling location were marked. As for Figure 4B, to batter explain it, we added Table S3 as supporting information.

Fig. 3B, it is impossible to distinguish so many colors in the figure. May use abbreviations to label different groups. It’s hard to know what the labels in the figure means. Need some explanation in figure legend.

**-Response:** This figure has been revised according to your suggestion. The explanation for the labels in the figure has been added in figure legends.

Fig. 4B, remove “k=1” part. How are the samples ordered? By varietas and by positions they were collected? Any further explanation about the observation? Are samples collected in the same places more likely to have similar genetic contents? Did you get consistent result if running admixture multiple times with different random seed numbers?

**-Response:** Thank you for your comments. Samples were ordered according to the genetic diversity. Most of the samples from the same place more likely to have similar genetic contents. In the data process, we carried out the structure analysis results according to the results of linkage disequilibrium attenuation analysis. Consistent result was got with different random seed numbers.

Fig. 5, I think it is better to plot 2D figures, and try to connect the results in Fig. 4B with the PCA result. Some samples from the varietas of A.fistulosum L. var. viviparum Makino are very close to A.fistulosum L. var. gigantum Makino but far from other samples from the same varietas. Any explanation?

**-Response:** The 2D PCA figure was added according to your suggestion. Only three groups were identified in the 2D figure, and then the related text was revised. The correlation with Figure 4B was explanted and analyzed then.

Line 32, Principal component analysis, not “Principal components analysis”.

**-Response:** Done as suggested.

Line 105, add citation for SOAP software.

**-Response:** Reference Li et al 2009 was cited according to your suggestion.

Line 157, “Group 1, 2 and 3 respectively included 11, 14 and 97 accessions”. So the 122 accessions includes 107 A.fistulosum L. var. viviparum Makino, 3 A.fistulosum L. var. gigantum Makino, 3 A.mongolicum Regel and 9 A.cepa L. accessions. What samples are in these three groups? Any relationship with the species?

**-Response:** Thank you for your suggestions, we reversed this part as suggested.

**Reviewer #2:**

The manuscript: “Genome-wide assessment of population structure and genetic diversity of Chinese Lou onion using specific length amplified fragment (SLAF) sequencing” is well written. The main contribution deals with the analysis of 122 A. fistulosum accessions were, as expected, regional and varietal genetic diversity were found. Also is valuable the use of the SLAF approach.

However, the biological and agronomical point of view is poorly discussed. In table 1, the authors have many data that can be discussed and they only mention that the accessions from the two sites at Tongxin, Ningxia, had relative higher dry matter rate and firmness, plant weight and height, pseudostem weight, length and diameter, and follow height. However, there was not difference in the pseudostem index. They do not relate these differences with the molecular data. They authors also mention that SNP-based markers could be used for the construction of high-density genetic map, linkage map and quantitative trait loci (QTL) mapping, which is true as long as they have segregating populations to perform this analysis.

**-Response:** Thank you very much for your kindly suggestions. In order to better express the views and conclusions of this manuscript, we revised the discussion section. The combination of different evolutionary relationships and traits were discussed.

Some other comments:

Key words: I suggest eliminating genetic evolution, and including Allium evolution

**-Response:** Done as suggested.

Please review the systematics, indeed A. fistulosum is an Amaryllidaceae, but also an Allioideae (former Alliaceae). The authors should carefully review the systematics of the specie.

**-Response:** Thanks for your suggestion. We changed it to “, …. also known as Chinese red onion, the Amaryllidaceae family and Allium genus (Allium, Allioideae, Amaryllidaceae”. The subfamily Allioideae is often disregarded in our mind. Thanks for your reminding.

Instead of the title Determination of the biophysical traits, I suggest to include Agronomic Traits

**-Response:** Thanks for your suggestions. The inclusion of agronomic traits may increase the novelty of our paper. We determined the agronomic traits of accessions from 8 sites in Ningxia, not all the 23 sites in China. There was no consistency with the sequence analysis.

I also recommend the author to review papers published in The Allium Genomes (2018) edited by Dr. Masayoshi Shigyo, published by Springer.

**-Response:** Thanks for our recommendation. However, the Dr. Masayoshi Shigyo published The Allium Genomes based on the Allium Species from Middle Asia. The “Taxonomical and Ethnobotanical Aspects of Allium Species from Middle Asia” and “Classical Genetics on Gene Mapping” sections were cited.

In compliance with data protection regulations, you may request that we remove your personal registration details at any time.  [(Remove my information/details)](https://www.editorialmanager.com/pone/login.asp?a=r). Please contact the publication office if you have any questions.

**-Response:** We tried our best to improve the manuscript and made some changes in the manuscript according to your suggestions. These changes will not influence the content and substance of the paper. And here we did not list the changes but *marked in a Tracked Changes version* in revised paper.

Once again, thank you very much for your comments and suggestions

Sincerely,

Dr. Tianhai Fang
