# Supplementary material for: Genome-wide assessment of population structure and genetic diversity of Chinese Lou onion using specific length amplified fragment (SLAF) sequencing
Source: PLoS One. 2020 May 5;15(5):e0231753. doi: 10.1371/journal.pone.0231753 (PMC7199963; doi:10.1371/journal.pone.0231753)
Supplement: Attachment — Submitted filename: Response to reviewers-2nd.docx [file pone.0231753.s005.docx]

Dear Tzen-Yuh Chiang,

Academic Editor

PLOS ONE,

We are responding to the further comments from editor and reviewers concerning our manuscript entitled “Genome-wide assessment of population structure and genetic diversity of Chinese Lou onion using specific length amplified fragment (SLAF) sequencing” (PONE-D-19-22783R1).

Thank you for your letter and for the comments. We have studied the comments carefully and have addressed them carefully.

**Point-by-point response to the reviewer’s comments**

PONE-D-19-22783R1
Genome-wide assessment of population structure and genetic diversity of Chinese Lou onion using specific length amplified fragment (SLAF) sequencing
PLOS ONE

[Note: HTML markup is below. Please do not edit.]

Reviewers' comments:

Reviewer's Responses to Questions

**Comments to the Author**

1. If the authors have adequately addressed your comments raised in a previous round of review and you feel that this manuscript is now acceptable for publication, you may indicate that here to bypass the “Comments to the Author” section, enter your conflict of interest statement in the “Confidential to Editor” section, and submit your "Accept" recommendation.

Reviewer #1: All comments have been addressed
Reviewer #2: All comments have been addressed

2. Is the manuscript technically sound, and do the data support the conclusions?
The manuscript must describe a technically sound piece of scientific research with data that supports the conclusions. Experiments must have been conducted rigorously, with appropriate controls, replication, and sample sizes. The conclusions must be drawn appropriately based on the data presented.

Reviewer #1: No

Reviewer #2: Yes

Response: The comments of the first reviewer on our revision and data was very professional and profound. We responded the main questions on discordant parts following questions (Comment 1).

3. Has the statistical analysis been performed appropriately and rigorously?

Reviewer #1: No

Reviewer #2: Yes

Response: Does the first review mean the discordant parts in data? I look forward to receiving your criticism upon your specific comments, if there are any questions on the methods of statistical analysis.

4. Have the authors made all data underlying the findings in their manuscript fully available?

The [PLOS Data policy](http://www.plosone.org/static/policies.action#sharing) requires authors to make all data underlying the findings described in their manuscript fully available without restriction, with rare exception (please refer to the Data Availability Statement in the manuscript PDF file). The data should be provided as part of the manuscript or its supporting information, or deposited to a public repository. For example, in addition to summary statistics, the data points behind means, medians and variance measures should be available. If there are restrictions on publicly sharing data—e.g. participant privacy or use of data from a third party—those must be specified.

Reviewer #1: Yes

Reviewer #2: Yes

5. Is the manuscript presented in an intelligible fashion and written in standard English?

PLOS ONE does not copyedit accepted manuscripts, so the language in submitted articles must be clear, correct, and unambiguous. Any typographical or grammatical errors should be corrected at revision, so please note any specific errors here.

Reviewer #1: Yes

Reviewer #2: Yes

6. Review Comments to the Author

Please use the space provided to explain your answers to the questions above. You may also include additional comments for the author, including concerns about dual publication, research ethics, or publication ethics. (Please upload your review as an attachment if it exceeds 20,000 characters)

Reviewer #1: The authors made some improvements based on the comments. But the manuscript and the response from the authors still hold some errors which are too obvious. Based on the description and the original data provided by the author, I have to draw the conclusion that the data in this manuscript is not reliable. Thus, I suggest to reject this manuscript.

**Response：**Thanks for your comments. Your confusion might due to the inconsistency between the number of accessions used for description of biophysical traits (69 accessions originated from 23 sites) and molecular study (a total of 122 accessions originated from 23 sites and 5 out-groups). The description of biophysical traits was only performed on 69 accessions from 23 sites. It is well know that the number of samples in each group should be the same at the time of statistics. Since only three samples can be collected in some regions, we choose a sample size of 3 (n=3) for statistics. However, the number of samples does not affect the construction of evolutionary trees, so we used all the 122 samples in the phylogenetic analysis. Besides, the accessions of A.cea L. accessions (Red, 24-1, 24-2, and 24-3), A.cea L. accessions (White, 25-1, 25-2, and 25-3), A.cea L. accessions (Yellow, 26-1, 26-2, and 26-3), A.var.gigantum (27-1, 27-2, and 27-3), and A.mongolicum Regel (28-1, 28-2, and 28-3) were used as out-groups in the phylogenetic analysis, there is no need to investigate their agronomic traits.

The accession number is not “subCRA002329”.**Response：**Thanks for your comments. The submitted accession number is CRA002156, not “subCRA002329”.

Fig. 2 is already removed. So the figure legends and figure numbers in the manuscript are wrong.

**Response：**Thanks for your comments. We forgot to submit Fig. 2. It has been uploaded this time.

The major concern is the discordant of Table 1 and Table S1. It is hard to allow such errors. In table 1, as the sample names are missing in table S1, we have to count number of samples. However, “Datong, Qinghai”, sample names are “1-1, 1-2, 1-3, 1-4, 1-5” which include 5 samples, but there are only 3 samples in table S1. “Zhongwei,Ningxia” samples are “29-1, 29-2, 29-3”, but the indexes are “16” in table S1 for that site. In fact, there are too many discordant parts in these two tables.

Response: Sorry for the inadequate description in table 1. We had added the sample number in revised MS. And we renamed the ‘29-1’, ‘29-2’, and ‘29-3’ to ‘6-1’, ‘6-2’, and ‘6-3’, respectively (There was no 6 in the original group name, but 29). So, there should be no misunderstanding. The sample number in Table S1 and S2 have been revised accordingly. In addition, the count number of accessions used for sequencing was 3-5 (a total of 122 accessions originated from 23 sites and 5 out-groups). Correspondingly, we modified the Figure 3, and we labeled the site locations and sample ID in Figure 3. For biophysical traits analysis, we used 3 individuals for each site (69 accessions originated from 23 sites, n=3). The discordant parts between the two tables have been addressed as above. We have reordered the tables (the numbers are not changed) to make it easier to read. In addition, we also marked the sample number n = 3 in Table 1.

We thank you very much for your hard work and patience with our paper. We hope the correction and responses to the comments met your approval.

**Reviewer #2**: All the suggestions made in the previous review had been incorporated. The manuscript has improved a lot and can be published.

Response: We thank you very much for your hard work and patience with our paper.

7. PLOS authors have the option to publish the peer review history of their article ([what does this mean?](https://journals.plos.org/plosone/s/editorial-and-peer-review-process#loc-peer-review-history)). If published, this will include your full peer review and any attached files.


If you choose “no”, your identity will remain anonymous but your review may still be made public.
**Response： No.**

Once again, thank you very much for your comments and suggestions

Sincerely,

Dr. Fang
